# Gene expression signature based screening identifies ribonucleotide reductase as a candidate therapeutic target in Ewing sarcoma

**DOI:** 10.18632/oncotarget.11416

**Published:** 2016-08-19

**Authors:** Kelli L. Goss, David J. Gordon

**Affiliations:** ^1^ Department of Pediatrics, Division of Pediatric Hematology/Oncology, University of Iowa, Iowa City, Iowa, USA

**Keywords:** Ewing sarcoma, ribonucleotide reductase, ciclopirox, iron chelator

## Abstract

There is a critical need in cancer therapeutics to identify targeted therapies that will improve outcomes and decrease toxicities compared to conventional, cytotoxic chemotherapy. Ewing sarcoma is a highly aggressive bone and soft tissue cancer that is caused by the EWS-FLI1 fusion protein. Although EWS-FLI1 is specific for cancer cells, and required for tumorigenesis, directly targeting this transcription factor has proven challenging. Consequently, targeting unique dependencies or key downstream mediators of EWS-FLI1 represent important alternative strategies. We used gene expression data derived from a genetically defined model of Ewing sarcoma to interrogate the Connectivity Map and identify a class of drugs, iron chelators, that downregulate a significant number of EWS-FLI1 target genes. We then identified ribonucleotide reductase M2 (RRM2), the iron-dependent subunit of ribonucleotide reductase (RNR), as one mediator of iron chelator toxicity in Ewing sarcoma cells. Inhibition of RNR in Ewing sarcoma cells caused apoptosis *in vitro* and attenuated tumor growth in an *in vivo*, xenograft model. Additionally, we discovered that the sensitivity of Ewing sarcoma cells to inhibition or suppression of RNR is mediated, in part, by high levels of SLFN11, a protein that sensitizes cells to DNA damage. This work demonstrates a unique dependency of Ewing sarcoma cells on RNR and supports further investigation of RNR inhibitors, which are currently used in clinical practice, as a novel approach for treating Ewing sarcoma.

## INTRODUCTION

Ewing sarcoma is an aggressive solid tumor that is treated with highly intensive, cytotoxic chemotherapy in combination with surgery and/or radiation [1]. Ewing sarcoma tumors are defined by a recurrent chromosomal translocation between the *EWSR1* gene and various *ETS* genes; the most common fusion, EWS-FLI1, is present in 85% of cases [2]. The EWS-FLI1 oncoprotein functions, in part, as an aberrant transcription factor and drives the expression of a set of genes that is oncogenic in a permissive cell context [3]. EWS-FLI1 is an attractive therapeutic target in Ewing sarcoma tumors because it is both required for tumorigenesis and specific for tumor cells [4–8]. However, directly targeting a transcription factor is difficult and EWS-FLI1 has proven to be a challenging target. Consequently, an alternative therapeutic strategy in treating Ewing sarcoma is to identify downstream targets, or unique dependencies, of EWS-FLI1 [6, 9–18].

The identification of novel targets in Ewing sarcoma is complicated by the observation that the effects of EWS-FLI1, including its impact on gene expression, are highly dependent on the cellular background [19]. Consequently, a number of different model systems, utilizing both gain-of-function and loss-of-function approaches, have been developed in a wide variety of cell types to identify the downstream targets of EWS-FLI1. Although some target genes are conserved across multiple models, there are also significant differences between the gene sets identified using these different experimental approaches and cellular backgrounds. Hancock et al. used a meta-analysis approach with 13 independent data sets to address this heterogeneity and identify a ‘core EWS-FLI1 gene expression signature [19].’ Similarly, Kauer et al. used multiple experimental approaches to identify a consensus list of genes regulated by EWS-FLI1 [20]. Despite these efforts, the overlap between these lists is modest and the transcriptional impact of EWS-FLI1 remains an active area of investigation.

In this study, we used gene expression data from an inducible, genetically defined model of Ewing sarcoma, which we recently developed using human embryonic stem cells, to identify a set of EWS-FLI1 target genes [21]. We then used this gene set to query the Connectivity Map (Broad Institute), a computational resource that identifies links between drugs and gene expression signatures, and identify a class of drugs, iron chelators, that downregulate genes that are upregulated by EWS-FLI1 [22]. We then identified ribonucleotide reductase M2 (RRM2), the iron-dependent subunit of ribonucleotide reductase (RNR), as one mediator of iron chelator toxicity in Ewing sarcoma cells [23]. Treatment of Ewing sarcoma cells with ciclopirox, as well as other drugs and siRNA that target RNR, induces apoptosis. In additional work, we discovered that high levels of SLFN11, a protein that sensitizes cells to drugs that cause DNA damage, is partially responsible for the toxicity of the RNR inhibitors toward Ewing sarcoma.

## RESULTS

### Connectivity Map analysis identifies iron chelators as drugs that downregulate genes that are upregulated by EWS-FLI1

**Figure 1:**
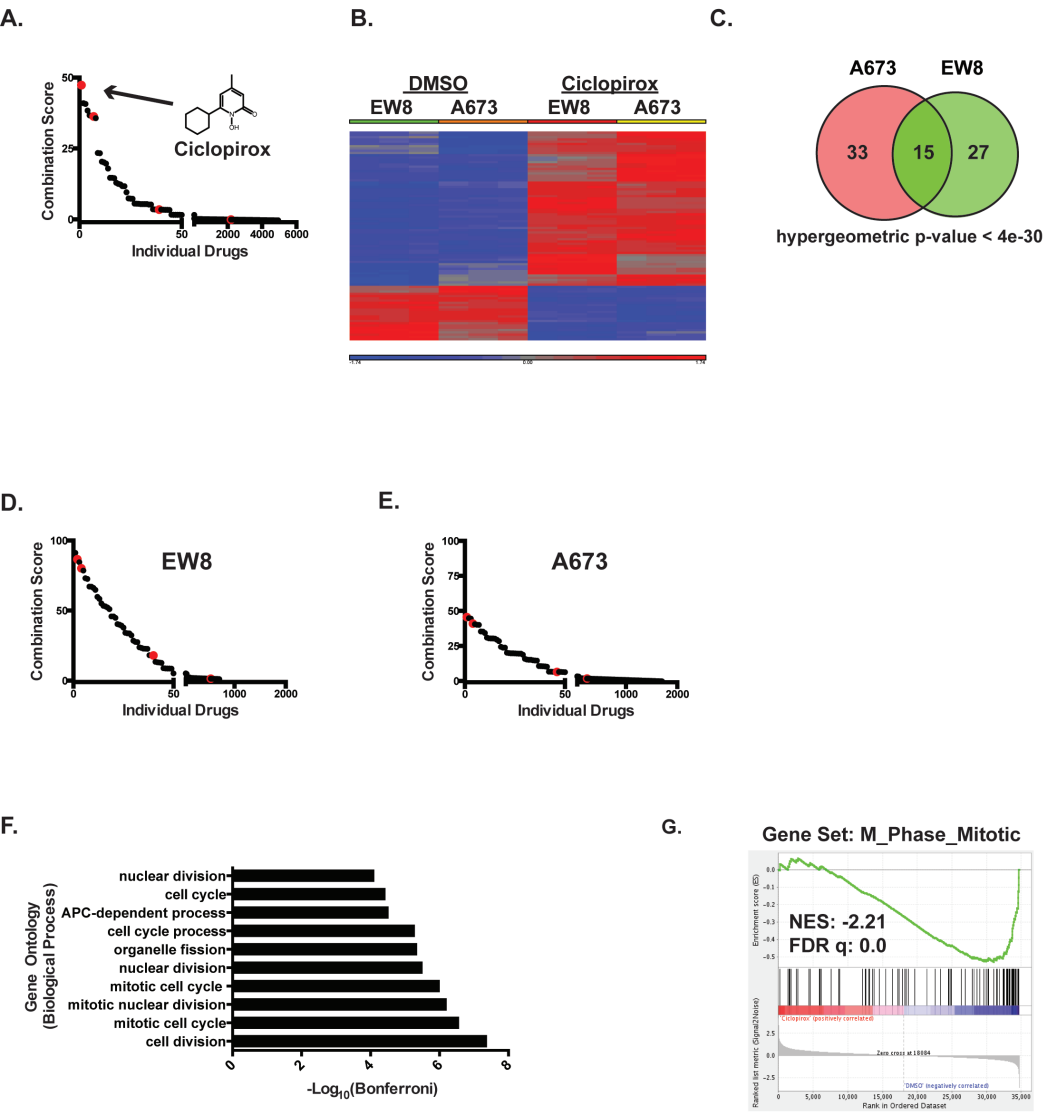
Identification of iron chelators as drugs that downregulate genes that are upregulated by EWS-FLI1 **A.** The Connectivity Map (CMAP) was used to identify drugs that downregulate genes that are upregulated by EWS-FLI1. The combined score, which is the product of the adjusted p-value and the z-score, is shown for the individual small molecules in CMAP. The red dots represent different cell lines (MCF7, PC3, HL60 and SKMEL5) treated with ciclopirox. Enrichment was calculated using the Enrichr resource [24]. The chemical structure of ciclopirox is shown as in insert in the graph. **B.** Heat map shows alteration in mRNA expression in Ewing sarcoma cell lines, EW8 and A673, treated with ciclopirox (10 μM) or DMSO for 24 hours. **C.** Venn diagram demonstrates the significant overlap between genes downregulated by ciclopirox in the two Ewing sarcoma cell lines. **D.**, **E.** CMAP was used to identify drugs that downregulate genes downregulated by ciclopirox in EW8 **D.** and A673 **E.** cells. The red dots represent different cell lines treated with ciclopirox. **F.** Gene ontology analysis of the genes downregulated by ciclopirox in the Ewing sarcoma cell lines was performed using ToppGene. **G.** Gene set enrichment analysis of expression data for Ewing sarcoma cells treated with ciclopirox shows a negative correlation between the M_Phase_Mitotic gene set and ciclopirox. The normalized enrichment scores (NER) and FDR q-values are shown.

In previous work, we developed an isogenic, inducible and reversible system to model the initiation of Ewing sarcoma in human embryonic stem cells [21]. We used this model system to identify 446 genes that are upregulated (Fold > 3 and FDR < 0.01), directly or indirectly, by the expression of the EWS-FLI1 oncoprotein (Supplementary Table 1). We then used Enrichr (http://amp.pharm.mssm.edu/Enrichr/) to interrogate the Connectivity Map (Broad Institute) and identify drugs that downregulate these EWS-FLI1 target genes [24]. We chose to focus on drugs that downregulate genes that are upregulated by EWS-FLI1 because many of these genes, including NR0B1, NKX2-2, CCND1, BCL11B, EZH2, are critical for tumorigenesis [11, 25–27]. Seventeen drugs demonstrated a gene expression signature with significant (adjusted *p*-value < 0.05) overlap with the EWS-FLI1 signature (Supplementary Table 2). Notably, three of these drugs are well-described iron chelators. The top hit identified in the analysis was ciclopirox (adjusted *p*-value = 8.22e-17), which is an iron chelator that is used for the treatment of topical fungal infections (Figure 1A). The second hit, 5109870 (ChemBridge), is also an iron chelator [28]. Finally, deferoxamine, an iron chelator that is used to treat iron overload, exhibited significant overlap with the EWS-FLI1 expression signature as well. In addition to iron chelators, CMAP analysis also identified etoposide, which is a critical component of current therapy for Ewing sarcoma, as a significant hit (adjusted *p*-value = 3.25e-10, combined score = 36.33). Similarly, resveratrol, which has been identified in other studies as a drug with potential therapeutic efficacy in Ewing sarcoma, was also identified in the analysis (adjusted *p*-value = 6.17e-11, combined score = 36.68) [29]. Additional, independent Ewing sarcoma gene expression data sets, which exhibit partial overlap with our gene set, also identified iron chelators as hits, although with lower enrichment scores (Supplementary Figure 1A-1C) [19, 20].

The CMAP gene expression data were generated using prostate, breast, melanoma, and leukemia cell lines [22]. To validate that ciclopirox causes similar gene expression changes in Ewing sarcoma cell lines, we performed gene expression microarray analysis on two Ewing sarcoma cell lines, A673 and EW8, treated with ciclopirox. As a comparison to the Ewing sarcoma cell lines, we also performed gene expression analysis on an osteosarcoma cell line, U2OS, and a telomerase-immortalized fibroblast cell line, BJ-tert. The cells were treated with ciclopirox, or vehicle, for 24 hours and then mRNA was collected for microarray gene expression analysis. Treatment of the Ewing sarcoma cells with ciclopirox resulted in the downregulation of ~45 genes in each of the cell lines (Fold > 2 and FDR < 0.05; Supplementary Table 3) (Figure 1B). There was significant (hypergeometric *p*-value < 4e-30) overlap in the downregulated genes between the Ewing sarcoma cell lines (Figure 1C). As predicted, CMAP analysis with these downregulated genes identified ciclopirox and 5109870 as significant hits (Figure 1D and 1E). Similar results were obtained using genes downregulated by ciclopirox in the U2OS and BJ-fibroblast cell lines, suggesting that some of the gene expression changes caused by ciclopirox are conserved between cell types (Supplementary Figure 1D-1F). Finally, the genes downregulated by ciclopirox in the Ewing sarcoma cell lines overlap significantly with genes upregulated by EWS-FLI1 (hypergeometric *p*-values < 8e-8 and 4e-12; Supplementary Figure 1G).

### Treatment of Ewing sarcoma cells with ciclopirox results in an accumulation of cells in S-phase

**Figure 2:**
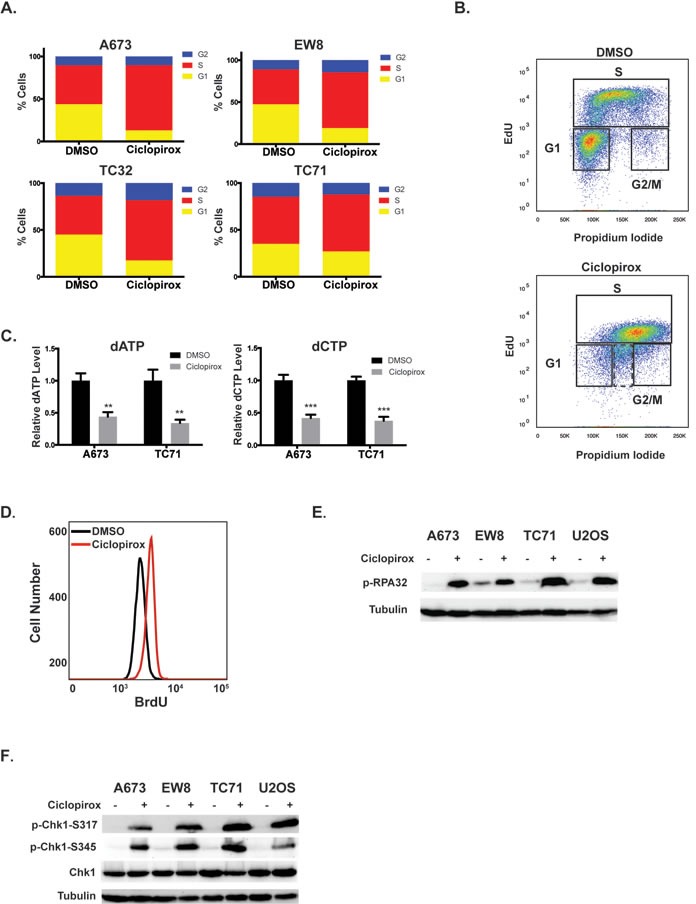
Ciclopirox impairs S phase progression of Ewing sarcoma cells **A.** Four Ewing sarcoma cell lines were treated with ciclopirox (10 μM) for 24 hours and then fixed to analyze their cell cycle distribution using propidium iodide. **B.** Cell cycle analysis with EdU and propidium iodide shows that treatment with ciclopirox (10 μM) results in a mixture of replicating and non-replicating (dotted box) S phase cells. **C.** Treatment of Ewing sarcoma cell lines with ciclopirox (10 μM) for 24 hours causes a reduction in dATP and dCTP levels. **D.** Quantification of ssDNA, using a non-denaturing BrdU assay, in cells treated with ciclopirox (10 μM). **E.**, **F.** Western blots showing that treatment of Ewing sarcoma cell lines with ciclopirox (10 μM) causes phosphorylation of RPA32 **E.** and Chk1 **F.**

The genes that are downregulated by ciclopirox in the Ewing sarcoma cells (Supplementary Table 4) are significantly (Bonferonni *p*-values < 1e-5 to 1e-8) enriched for cell cycle genes (Figure 1F) [30]. Similarly, gene set enrichment analysis (GSEA) of the DMSO- and ciclopirox-treated cell lines identified that multiple gene sets related to the cell cycle are negatively correlated with ciclopirox (FDR *q*-value = 0.0; Figure 1G) [31]. Based on this gene enrichment data, we used propidium iodide to test whether ciclopirox affects the cell cycle progression of Ewing sarcoma cells. Treatment of Ewing sarcoma cells with ciclopirox for 24 hours led to an accumulation of cells in S-phase (Figures 2A and Supplementary Figure 2). To determine if the S-phase cells were replicating DNA we performed dual labeling with propidium iodide and EdU. The dual labeling demonstrated that treatment of Ewing sarcoma cells with ciclopirox results in a mixture of replicating and non-replicating S-phase cells (Figure 2B). Additionally, the replicating S-phase cells exhibited reduced incorporation of EdU compared to the control cells.

Iron chelator drugs are known to inhibit DNA replication and block cell cycle progression in late G1 or S phase by inhibiting ribonucleotide reductase M2 (RRM2), the iron-dependent subunit of ribonucleotide reductase (RNR) [23, 32]. RNR catalyzes the formation of deoxyribonucleotides from ribonucleotides and inhibiting RNR, by targeting either the RRM1 or RRM2 subunit of the enzyme, impairs DNA replication and causes replication stress [23]. RRM2 is highly expressed in Ewing sarcoma cells compared to other cancer types (*p*-value < 0.01; Supplementary Figure 3) and treatment of Ewing sarcoma cells with ciclopirox resulted in a significant reduction in deoxyribonucleotide levels, as predicted if RRM2 is a target of ciclopirox (Figure 2C). Similarly, treatment of Ewing sarcoma cells with ciclopirox caused an increase in single-strand DNA (ssDNA), which is an indicator of impaired DNA replication (Figure 2D) [33]. Treatment of Ewing sarcoma cells with ciclopirox also resulted in the phosphorylation of replication protein A (RPA32), which associates with ssDNA and is a marker of replication stress (Figure 2E) [33]. We also detected phosphorylation of checkpoint kinase 1 (Chk1), the major regulator of the response to impaired DNA replication, after treatment with ciclopirox (Figure 2F) [34].

### Treatment of Ewing sarcoma cells with ciclopirox results in impaired growth and apoptosis

**Figure 3:**
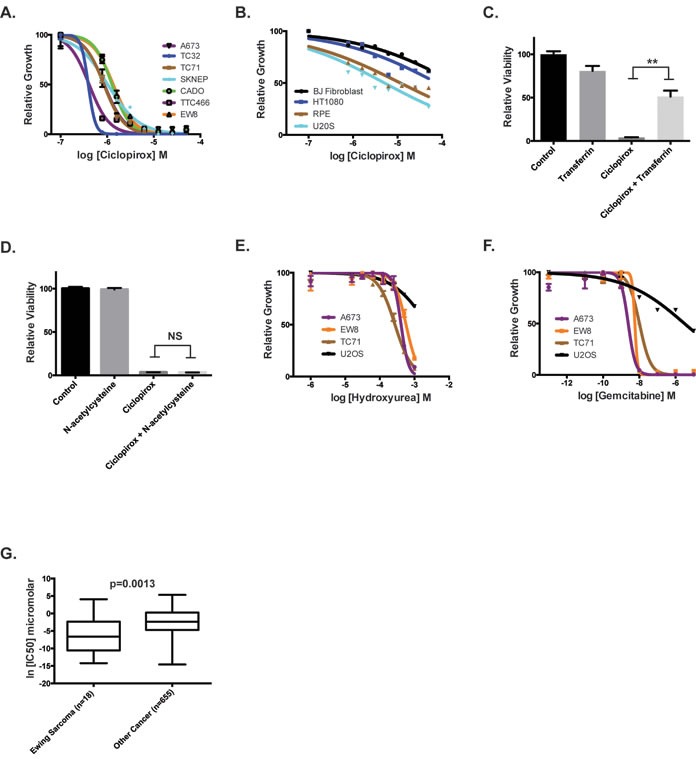
Inhibitors of ribonucleotide reductase impair the growth of Ewing sarcoma cells **A.** Dose-response curves for seven Ewing sarcoma cell lines treated with different concentrations of ciclopirox for three days. Cell viability was assessed using the CellTiter-Glo Luminescent Assay. **B.** Dose-response curves for non-Ewing sarcoma cell lines treated with different concentrations of ciclopirox for three days. **C.** Relative viability of A673 cells treated with transferrin, ciclopirox and the combination of ciclopirox and transferrin. **D.** Relative viability of A673 cells treated with N-acetylcysteine, ciclopirox and the combination of ciclopirox and N-acetylcysteine. **E.** Dose-response curves for Ewing sarcoma cell lines treated with different concentrations of hydroxyurea for three days. **F.** Dose-response curves for Ewing sarcoma cell lines treated with different concentrations of gemcitabine for three days. **G.** Analysis of Genomics of Drug Sensitivity in Cancer data shows that Ewing sarcoma cell lines are more sensitive to gemcitabine than other cancer cell lines. For the dose-response experiments, the results are representative of three independent experiments. Error bars represent mean ± SD of three technical replicates. ** *P*-value < 0.01.

Treatment of Ewing sarcoma cell lines with ciclopirox for 72 hours caused a significant reduction in growth (Figure 3A), with IC50 values ranging from 500 nM to 3 μM. In contrast, ciclopirox was less effective at inhibiting the growth of other cell lines, including HT1080 (fibrosarcoma), U2OS (osteosarcoma), BJ-tert (telomerase-immortalized fibroblasts) and RPE-tert (telomerase-immortalized epithelial cells) (Figure 3B). Treatment with the highest concentration of ciclopirox (50 μM) resulted in < 50% growth reduction in these cell lines. Ewing sarcoma cells were also sensitive to treatment with an additional iron chelator drug, deferasirox (Supplementary Figure 4A-4B). Notably, the addition of holo-transferrin, a source of biologically available iron, to the cell culture media significantly rescued the toxicity of ciclopirox toward Ewing sarcoma cells, demonstrating that iron is a target of ciclopirox (Figure 3C). However, the antioxidant N-acetylcysteine, in contrast to holo-transferrin, was unable to rescue the toxicity of ciclopirox, demonstrating that the effect of ciclopirox is not mediated by altering thiol-based redox homeostasis, as can be seen with other iron chelators (Figure 3D). Similarly, ciclopirox treatment did not have an effect on EWS-FLI1 levels (Supplementary Figure 5).

Next, we tested whether additional small molecule inhibitors of RNR could reduce the growth and viability of Ewing sarcoma cells. Hydroxyurea, a well-established inhibitor of RRM2, inhibited the growth of Ewing sarcoma cells at concentrations (IC50 range 165-300 μM) that are significantly lower than concentrations typically used for cell cycle synchronization (> 1 mM) (Figure 3E) [35]. Ewing sarcoma cells were also sensitive (IC50 range 2.4-10 nM) to gemcitabine, an inhibitor of RRM1 (Figure 3F). Furthermore, analysis of the Genomics of Drug Sensitivity in Cancer Project (http://www.cancerrxgene.org/) data, which includes 18 Ewing sarcoma cell lines and > 600 other cancer cell lines, demonstrated that Ewing sarcoma cells are significantly more sensitive to gemcitabine than other cancer types (*p*-value = 0.0013) (Figure 3G) [36].

**Figure 4:**
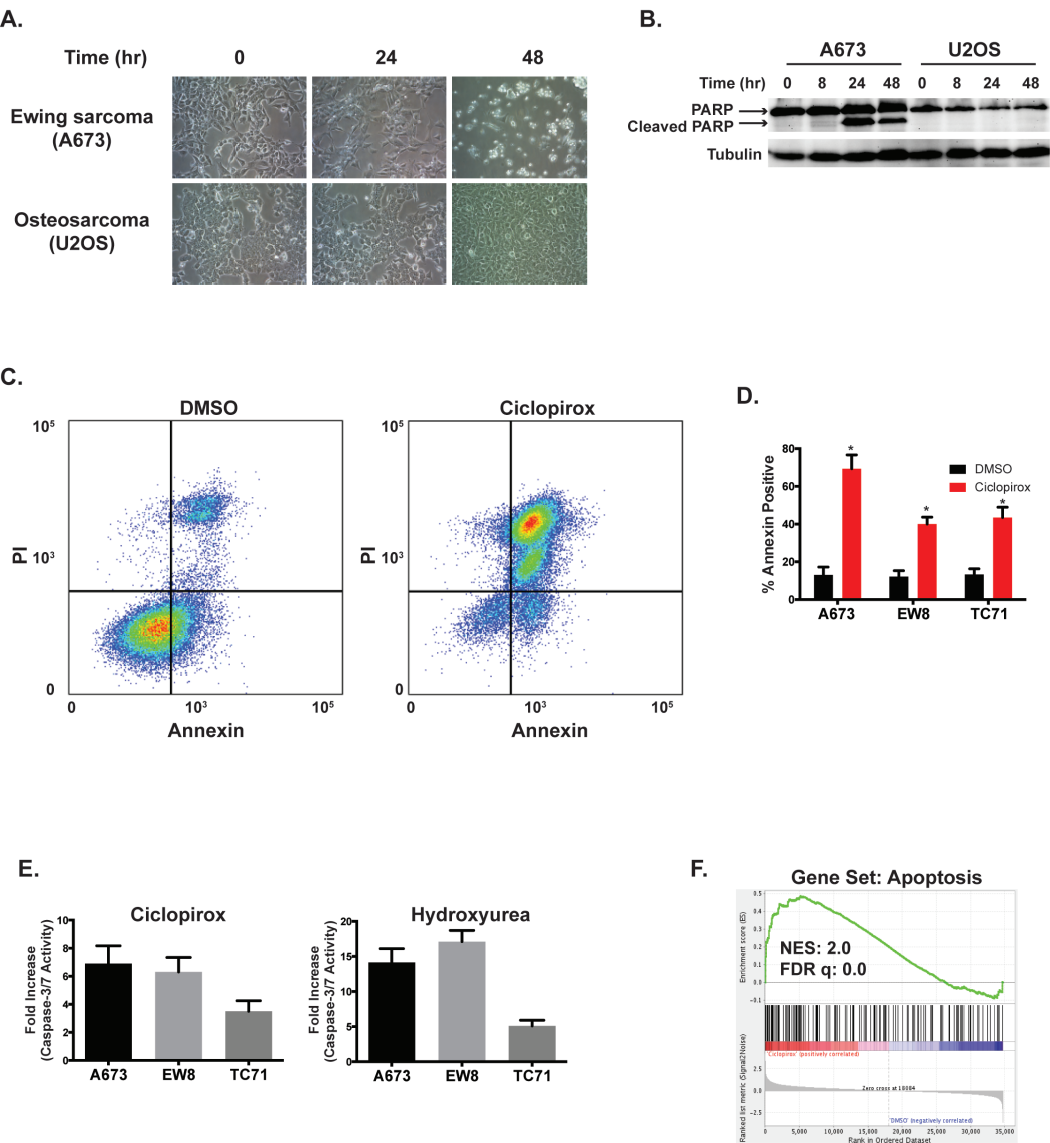
Ciclopirox induces apoptosis in Ewing sarcoma cells **A.** Treatment with ciclopirox (10 μM) causes morphologic changes in Ewing sarcoma cells. **B.** Western blot showing that treatment of Ewing sarcoma cells, but not U2OS osteosarcoma cells, with ciclopirox (10 μM) causes cleavage of PARP-1. **C.** Flow cytometry plot for Annexin and PI staining of A673 cells treated with DMSO or ciclopirox (10 μM) for two days. **D.** Percentage of Annexin-V positive cells for three Ewing sarcoma cell lines treated with ciclopirox (10 μM) for two days. Results are representative of two independent experiments. Error bars represent mean ± SD of two technical replicates. **E.** Fold increase in caspase-3/7 activation in three Ewing sarcoma cells lines treated with ciclopirox (10 μM) and hydroxyurea (500 μM) for three days. Fold change is relative to cells treated with DMSO. Figures are representative of three independent experiments. Data represent mean ± SD of three technical replicates. **F.** Gene set enrichment analysis of expression data for Ewing sarcoma cells treated with ciclopirox shows a correlation between the Apoptosis gene set and ciclopirox. The normalized enrichment scores (NER) and FDR q-values are shown. * *P*-value < 0.05.

In addition to the effects on cell cycle, we also noted significant morphologic changes, suggestive of cell death, in the Ewing sarcoma cells treated with 10 μM ciclopirox (Figure 4A), which is a drug concentration that significantly impairs the viability of all of the Ewing sarcoma cell lines (Figure 3B) and is an achievable serum concentration of the drug *in vivo* [32]. These morphologic changes coincided with cleavage of PARP-1, a marker of apoptosis (Figure 4B). Notably, an osteosarcoma cell line, U2OS, treated with ciclopirox did not demonstrate cleavage of PARP-1. Treatment of Ewing sarcoma cells with ciclopirox also resulted in an increase in the percentage of cells positive for annexin-V and propidium iodide (Figure 4C and 4D). Similarly, a luminescence-based assay (Caspase-Glo 3/7; Promega) demonstrated activation of caspase-3/7 in Ewing sarcoma cells treated with ciclopirox and hydroxyurea (Figure 4E). Gene set enrichment analysis of the DMSO- and ciclopirox-treated Ewing sarcoma cells also identified the upregulation of genes related to apoptosis in the cells treated with ciclopirox (Figure 4F).

### Treatment of Ewing sarcoma cells with siRNA targeting RNR results in impaired growth and apoptosis

**Figure 5:**
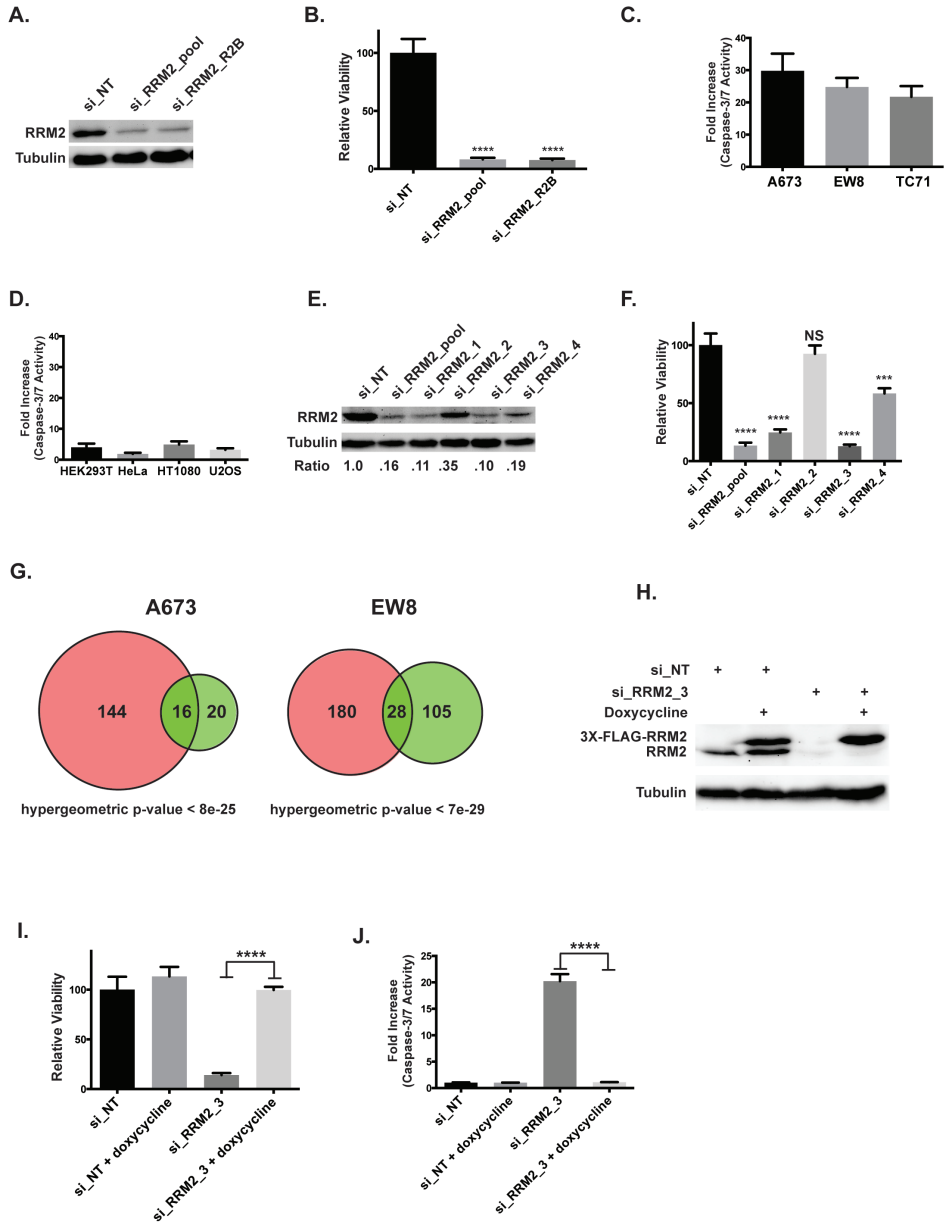
Knockdown of RRM2 using siRNA impairs the viability of Ewing sarcoma cells **A.** Western blot showing levels of the RRM2 protein in A673 cells after transfection with two different siRNAs targeting RRM2 or a non-targeting siRNA (si_NT). **B.** Relative viability of A673 cells treated with a non-targeting siRNA or the two siRNAs that target RRM2. **C.**, **D.** Increase in caspase-3/7 activation in Ewing sarcoma cells lines **C.** and non-Ewing sarcoma cell lines **D.** treated with siRRM2_R2B or si_NT for three days. Results are plotted as the fold increase in caspse-3/7 activity in the cells treated with siRRM2_R2B compared to the cells treated with si_NT. **E.** Western blot showing levels of the RRM2 protein after transfection with si_RRM2_pool or the individual siRNAs that compose the pool set. The relative expression level of RRM2 for each siRNA compared to the non-targeting siRNA is shown below the plot. **F.** Relative viability of A673 cells transfected with si_NT, si_RRM2_pool and the individual siRNAs that compose the pool (1-way ANOVA, Dunnett's *post hoc* test) **G.** Venn diagram demonstrates the overlap between genes deregulated by ciclopirox (pink) and si_RRM2_R2B (green). **H.** Western blot showing that the exogenous, doxycycline-inducible RRM2 gene, which is labeled with a 3X-FLAG tag, is resistant to knockdown by si_RRM2_3. **I.** Relative viability of EW8 cells with doxycycline-inducible expression of siRNA-resistant RRM2 after transfection with si_RRM2_3. Cell viability was assessed using the CellTiter-Glo Luminescent Assay. **J.** Increase in caspase-3/7 activation in EW8 cells with doxycycline-inducible expression of siRNA-resistant RRM2 after transfection with si_RRM2_3. Fold change is relative to the si_NT cells. Data **B.**, **C.**, **D.**, **F.**, **I.**, **J.** represent mean ± SD, *n* = 3. *** *p* -value < 0.001, **** *p* -value < 0.0001.

We then used siRNA to knockdown RRM2 in Ewing sarcoma cells to complement the small-molecule studies. Two different siRNAs, a “pool” set consisting of four unique siRNAs (si_RRM2_pool) and a well-validated siRNA used in clinical trials (si_RRM2_R2B), were used to knockdown RRM2 (Figure 5A) [37, 38]. Knockdown of RRM2 with siRNA resulted in a significant reduction in cell growth (Figure 5B) and activation of caspase-3/7 (Figure 5C) in Ewing sarcoma cells, but not the other cell lines we tested (Figure 5D). Deconvolution of the si_RRM2_pool set showed that siRRM2_1 and siRRM2_3 were most effective at depleting RRM2 (Figure 5E) and that knockdown efficiency correlated with growth inhibition (Figure 5F). To test the effects of RRM2 knockdown on gene expression, we performed gene expression analysis on A673 and EW8 cells treated with si_RRM2_R2B and a non-targeting siRNA (si_NT). There was significant overlap in the genes deregulated by ciclopirox and si_RRM2_R2B (hypergeometric *p*-values < 8e-25 and < 7e-29), as predicted if RRM2 is a target of ciclopirox (Figure 5G). More genes were deregulated by ciclopirox than the RRM2 siRNA, which likely reflects off-target effects of the drug. Finally, to exclude siRNA off-target effects, we generated an Ewing sarcoma cell line that expresses a RRM2 gene, under the control of a doxycycline-inducible promoter, that is resistant to siRNA knockdown (Figure 5H). Notably, expression of this siRNA-resistant RRM2 gene in Ewing sarcoma cells rescued the growth defect (Figure 5I) and caspase-3/7 activation (Figure 5J) caused by transfection of si_RRM2_3 and knockdown of endogenous RRM2.

We also used a set of siRNAs targeting RRM1 (si_RRM1_pool), the other subunit of RNR, to deplete RRM1 in Ewing sarcoma cells. These siRNAs were specific for RRM1 and did not affect levels of RRM2 (Supplementary Figure 6A). All four siRNAs in the pool showed effective knockdown of RRM1 (Supplementary Figure 6B) and significant inhibition of Ewing sarcoma cell growth (Supplementary Figure 6C).

### SLFN11 contributes to the sensitivity of Ewing sarcoma cells to inhibition of RNR

**Figure 6:**
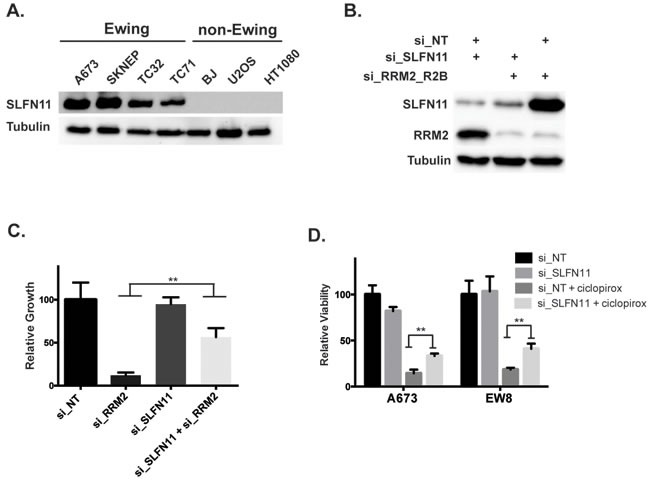
Elevated levels of SLFN11 contribute to the sensitivity of Ewing sarcoma cells to inhibition of RNR **A.** Western blot showing levels of SLFN11 in Ewing sarcoma cells and non-Ewing sarcoma cell lines. **B.** Western blot showing levels of SLFN11 and RRM2 after transfection of cells with siRNAs that target SLFN11, RRM2 or both SLFN11 and RRM2. **C.** Concurrent knockdown of RRM2 and SLFN11 with siRNA partially rescues the effects of RRM2 knockdown on Ewing sarcoma cell viability. **D.** Knockdown of SLFN11 with siRNA in Ewing sarcoma cells partially rescues cells from the toxicity of ciclopirox. ** *P*-value < 0.01; *** *P*-value < 0.001.

The Ewing sarcoma cells were more sensitive to RNR inhibition and knockdown than the other cell lines we tested. Notably, Ewing sarcoma cell lines are also known to be sensitive to PARP-1 inhibitors and combinations of PARP-1 inhibitors with DNA-damaging agents. This sensitivity is mediated, in part, by high levels of SLFN11, which is a putative helicase and a direct transcriptional target of EWS-FLI1 that is highly expressed in Ewing sarcoma tumors [39, 40]. Since SLFN11 is known to sensitize cancer cells to a number of drugs that cause DNA-damage, including DNA synthesis inhibitors, we tested the hypothesis that the sensitivity of Ewing sarcoma cells to RNR inhibitors may be caused by elevated levels of SLFN11 [39, 41]. Figure 6A demonstrates that SLFN11 is highly expressed in Ewing sarcoma cell lines, as previously reported [39]. To test whether SLFN11 expression modulates the sensitivity of Ewing sarcoma cells to RRM2 knockdown we used siRNA to knockdown both SLFN11 and RRM2 in Ewing sarcoma cells (Figure 6B). Notably, the toxicity from knockdown of RRM2 was partially rescued by co-knockdown of SLFN11 (Figure 6C). Additionally, knockdown of SLFN11 also partially rescued Ewing sarcoma cells from the effects of ciclopirox on cell viability (Figure 6D).

### Ewing sarcoma xenografts respond to ciclopirox

**Figure 7:**
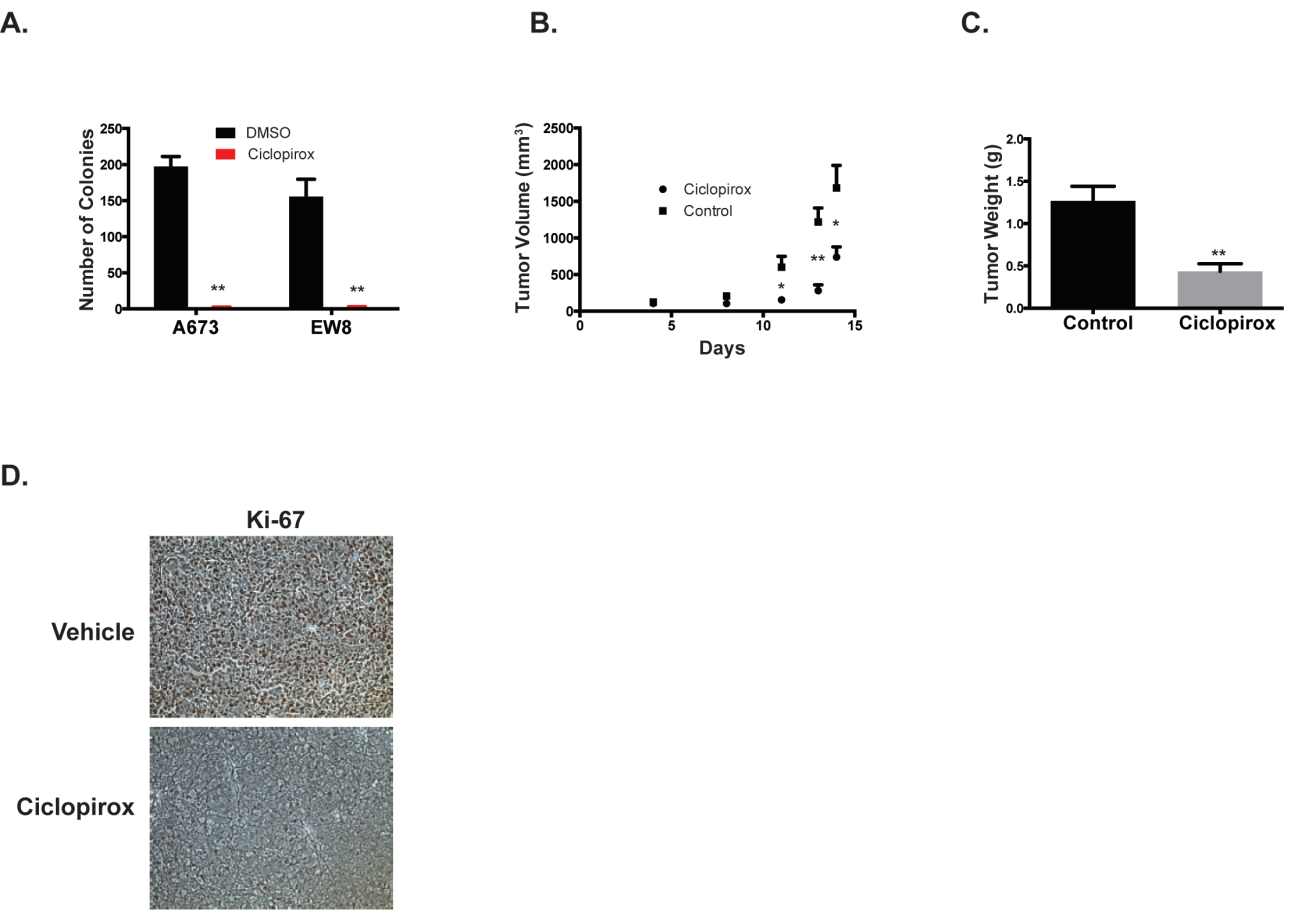
Ciclopirox inhibits the growth of Ewing sarcoma cells in suspension and in xenograft experiments **A.** Growth of Ewing sarcoma colonies in methylcellulose-based media treated with ciclopirox (10 μM) *versus* DMSO. Results are representative of two independent experiments. Error bars represent mean ± SD of three technical replicates. **B.**, **C.** A673 cells were engrafted in nude mice and treated by gavage with either vehicle or ciclopirox (25 mg/kg) (*n* = 10 mice per group). Tumor size during treatment **B.** and tumor weight at the end point **C.** are shown. **D.** Immunohistochemical staining for proliferation marker Ki-67. The xenograft data are representative of two independent experiments. * *P*-value < 0.05, ** *P*-value < 0.01.

Based on the *in vitro* growth inhibition and apoptosis data, we next tested whether ciclopirox could inhibit the growth of tumor cells in suspension and in mouse xenograft experiments. Ciclopirox significantly inhibited the growth of Ewing sarcoma cells in an anchorage-independent growth assay (Figure 7A). For the xenograft experiment, NCr mice were subcutaneously injected with A673 cells and allowed to develop measurable tumors. The mice were then treated with oral ciclopirox (25 mg/kg) or vehicle. Treatment with ciclopirox significantly decreased tumor size (Figure 7B). When the largest tumor in the control group reached ~2000 mm, all of the animals were euthanized and the tumors were excised and weighed. Ciclopirox significantly decreased tumor weight (Figure 7C). Ki-67 staining, a marker of cell proliferation, was also decreased in tumors from animals treated with ciclopirox (Figure 7D).

## DISCUSSION

Despite aggressive therapy, the overall survival of patients with metastatic and non-metastatic Ewing sarcoma are ~20% and ~70%, respectively [1]. Moreover, the current treatment of Ewing sarcoma, which consists of cytotoxic chemotherapy in combination with surgery and/or radiation, is associated with significant on- and off-treatment morbidities. EWS-FLI1 is an appealing target in Ewing sarcoma tumors and the identification of direct inhibitors of this transcription factor is an active area of investigation [5–7, 19, 42]. Other work has focused on identifying downstream targets, or unique vulnerabilities, of EWS-FLI1 [9–12, 15, 43, 44]. In this work, we used gene expression data from a genetically defined model of Ewing sarcoma to query the Connectivity Map and identify that Ewing sarcoma cells are sensitive to chemical inhibition and siRNA suppression of RNR. We used a doxycycline-inducible, siRNA-resistant, RRM2 transgene to demonstrate that the reduced viability and induction of apoptosis caused by siRNA knockdown of RRM2 is an on-target effect. We also identified that the elevated level of SLFN11 in Ewing sarcoma cells is responsible, in part, for the sensitivity of this sarcoma toward RNR inhibitors. Additionally, because multiple inhibitors of RNR are currently used in clinical oncology, we show that ciclopirox inhibits the *in vivo* growth of Ewing sarcoma cells in a xenograft model.

Ciclopirox is a small-molecule inhibitor of RNR that targets the iron center of the RRM2 subunit through an iron-chelation mechanism [32]. Although ciclopirox was originally developed as a topical antifungal, oral ciclopirox was recently shown to display biological activity in a phase I trial in adult patients with advanced hematologic malignancies [45]. A potential limitation of oral ciclopirox, though, is that it is subject to first-pass metabolism and has a short half-life *in vivo*, consistent with the modest effect that we observed in the xenograft experiment [45]. However, from a clinical standpoint, other inhibitors of RNR with more favorable pharmacokinetics are used extensively in clinical oncology [46].

RRM1 can be targeted using both allosteric inhibitors (fludarabine and clofarabine) and catalytic inhibitors (cytarabine and gemcitabine) [47]. Similarly, iron chelators, (ciclopirox, triapine and deferoxamine) and free radical scavengers (hydroxyurea) inhibit RRM2 [47]. The dimerization of RRM1 and RRM2 can also be blocked using small peptides and small molecules, such as COH29 [48]. Although small-molecule inhibitors represent the primary strategy for RNR inhibition, siRNA-based approaches to target RNR are also currently being tested in clinical trials [37, 38]. Furthermore, *in vitro* and *in vivo* work has demonstrated synergy between RRM1 and RRM2 inhibitors [49]. RNR inhibitors are also synergistic with other classes of chemotherapy drugs, which is critical because single agent chemotherapy is rarely curative [32, 50, 51]. For example, single-agent olaparib, a PARP inhibitor being tested in clinical trials for Ewing sarcoma, has modest *in vivo* activity in xenograft experiments, similar to ciclopirox [52]. However, the combination of olaparib with temozolomide or irinotecan results in complete and durable remissions in xenograft experiments [53]. Clinical trials testing gemcitabine, in combination with docetaxel, in patients with Ewing sarcoma have shown variable efficacy, which may be related to differences in gemcitabine doses between the regimens [54, 55]. A clinical trial testing single-agent cytarabine in ten patients with relapsed or refractory Ewing sarcoma did not show efficacy [43]. However, *in vitro* work by Stegmaier et al. showed that cytarabine reduced EWS-FLI1 protein levels [56]. We did not observe a similar decrease in EWS-FLI1 levels in Ewing sarcoma cells treated with ciclopirox (Supplementary Figure 5), suggesting that the mechanism of cytarabine-induced toxicity may be different than iron chelators. Consequently, extrapolating the results from the clinical trial with cytarabine to other inhibitors of RNR may be challenging. Thus, we believe that further testing of RNR inhibitors, in particular drugs with improved pharmacokinetic properties and drugs in combination therapy, against Ewing sarcoma is supported by the wide availability of RNR inhibitors, the extensive clinical experience with this class of drugs, and the synergy between RNR inhibitors and other chemotherapy agents.

The overexpression of RRM2 can promote transformation and tumorigenesis *via* its cooperation with several oncogenes, but the overexpression of RRM1 has been shown to suppress malignant potential [57, 58]. Consequently, the roles of RRM1, RRM2 and RNR in tumorigenesis are complex [47]. However, inhibition or suppression of RNR in cancer cells is known to cause senescence [59–61]. For example, Aird et al. has shown that knockdown of RRM2 triggers aberrant DNA replication, activation of the DNA damage response and cellular senescence in primary cells and cancer cell lines, including melanoma and ovarian cancer [59–61]. In Ewing sarcoma cells, however, we show that inhibition of RNR leads to apoptosis. An aberrant response to DNA damage is well described in Ewing sarcoma tumors, although the mechanism is unclear [53].

The induction of apoptosis caused by RNR inhibitors in Ewing sarcoma cells is due, in part, to high levels of SLFN11. SLFN11 is a direct transcriptional target of EWS-FLI1 and overexpressed in Ewing sarcoma tumors [39, 40]. Recent work has shown that SLFN11 causes defects in checkpoint maintenance and homologous recombination repair [62]. Zoppoli et al. demonstrated that high levels of SLFN11 confer sensitivity of cancer cell lines to topoisomerase inhibitors, alkylating agents and DNA synthesis inhibitors, including gemcitabine [41]. Similarly, Tang et al. reported that SLFN11 is responsible for the high sensitivity of Ewing sarcoma cells to camptothecin and combinations of PARP inhibitors with temozolomide [39]. Notably, PARP inhibitors can cause replication stress by a “trapping” mechanism whereby the inhibitor stabilizes a PARP-DNA complex that interferes with DNA replication [63]. The SLFN11 data, in addition to providing a mechanistic explanation for the sensitivity of Ewing sarcoma cells to RNR inhibitors, also suggest that expression of this protein could function as a biomarker to predict drug response. It is important to note, however, that the rescue of drug toxicity by SLFN11 is only partial, which suggests that other pathways and proteins may play critical roles in modulating the response to RNR inhibitors. Notably, EWS-FLI1 has been implicated as a regulator of multiple aspects of the cellular response to genotoxic stress, although the mechanistic details remain to be elucidated [64]. In addition, haploinsufficiency of the *EWSR1* gene in Ewing sarcoma tumors may also contribute to an impaired response to DNA damage [65].

We showed that suppression of RRM2 and loss of EWS-FLI1 expression result in the deregulation of an overlapping set of genes. One possible explanation for this overlap is that EWS-FLI1 regulates RNR levels. In particular, the RRM2 subunit of RNR is expressed at high levels in Ewing sarcoma cells relative to other cancers (Supplementary Figure 3). In our isogenic system, we detected a 5-fold and 140-fold downregulation of RRM1 and RRM2 mRNA levels, respectively, after loss of EWS-FLI1 expression [21]. Kauer et al. also identified RRM2 as a gene regulated by EWS-FLI1 expression and Hancock et al. identified RRM1 in their meta-analysis [19, 20]. However, based on CHIP-seq experiments, EWS-FLI1 does not appear to directly regulate RRM1 or RRM2 expression levels, although effects on enhancers or other regulatory units cannot be excluded [66]. An alternative possibility is that EWS-FLI1 regulates RNR though an indirect mechanism. For example, EWS-FLI1 deregulates the activity of E2F transcription factors, which are critical regulators of the cell cycle and the primary regulators of RRM2 transcription [67]. However, because EWS-FLI1 targets multiple regulators of the cell cycle, including cyclin D1 and cyclin A1, it is difficult to conclude whether the changes in RRM2 expression after loss of EWS-FLI1 expression are a cause or consequence of the changes in the cell cycle [20, 68]. Consequently, future work will need to investigate the regulation of RNR expression and activity in Ewing sarcoma.

In summary, we used gene expression data, in conjunction with the Connectivity Map, to identify that Ewing sarcoma tumors are sensitive to chemical inhibition and siRNA suppression of RNR. Overall, our work supports further study of RNR inhibitors in the treatment of Ewing sarcoma. In particular, the availability of clinically used inhibitors of RNR suggests the potential for translation of this work to the clinic.

## MATERIALS AND METHODS

### Cell lines and culture

Cell lines were maintained at 37°C in a 5% CO atmosphere. The A673, TC32, TC71, SK-NEP, CADO, TTC466, and EW8 cell lines were kindly provided by Dr. Kimberly Stegmaier (Dana-Farber Cancer Institute, Boston, MA). The BJ-tert, HEK-293T, HT1080, RPE-tert, and U2OS cell lines were obtained from ATCC. Cells were grown in Dulbecco's Modified Eagle's Media (DMEM) supplemented with 10% FBS, 100 IU ml penicillin and 100 μg ml streptomycin. Cell lines were authenticated by DNA fingerprinting using the short tandem repeat (STR) method.

### Chemical compounds

Chemical compounds were purchased from Sigma (ciclopirox, gemcitabine, and hydroxyurea) and Selleck Chemical (deferasirox).

### Connectivity map analysis

We used Enrichr (http://amp.pharm.mssm.edu/Enrichr) to query the Connectivity Map (CMAP) [22, 24]. Enrichr, which extracts the top 100 and bottom 100 differentially expressed genes for each of the drugs in CMAP, uses three approaches to compute enrichment, including the Fisher exact test, a z-score ranking generated using random input gene lists and a combination of the Fisher exact test and z-score (combination score).

### Cell viability

Cell proliferation was measured using CellTiter-Glo (Promega). Approximately 2-5 × 10 cells were plated per well of a 96-well plate. Cells were treated with a range of drug concentrations for three days. Luminescence readings were obtained after adding the CellTiter-Glo reagent using a FLUOstar Omega microplate reader (BMG Labtech). IC50 values were then calculated using log-transformed and normalized data (GraphPad Prism 5.0).

### Gene expression

For the microarray experiments, RNA was collected from three biological replicates of cells treated with DMSO, ciclopirox (10 μM), si_NT, and si_RRM2_R2B using an RNeasy kit (Qiagen). The samples were then prepared for analysis and hybridized to HumanHT-12 v4 (Illumina) BeadChips by the Microarray Core at the University of Iowa. Partek Genomics Suite Version 6.6 was used to normalize the raw microarray data, preprocess the normalized data using default parameters and find differentially expressed probe sets. Gene set enrichment analysis (GSEA) was performed using the GSEA platform (www.broadinstitute.org/gsea) [31]. The gene expression files were deposited in the Gene Expression Omnibus (GEO) Repository under the accession number GSE79641. Venn diagrams were prepared using BioVenn (http://www.cmbi.ru.nl/cdd/biovenn/).

### Xenograft

The Institutional Animal Care and Usage Committee at the University of Iowa approved the animal studies. Approximately 1.0 × 10 A673 cells were mixed with 30% matrigel and injected subcutaneously into the flanks of 6-week old, female NCr mice. After tumors were palpable, mice were divided into two cohorts and treated daily with ciclopirox (25 mg/kg) or vehicle control by oral gavage. Tumor volumes were measured periodically using calipers (volume = 0.5 x length x width). All animals were sacrificed when the largest tumors in either the control or treatment groups reached 20 mmin any dimension. Tumors were then excised from all animals and weighed.

### Immunohistochemistry

Tumor xenografts were fixed in formalin for immunohistochemical staining. Immunostaining for Ki67 was conducted using a rabbit anti-human Ki67 antibody (D2H10, Cell Signaling, #9027, 1:400) on tumor sections. Staining was performed using the Vectastain ABC Kit (Vector Laboratories), according to the manufacturer's instructions.

### ssDNA quantification

ssDNA was quantified using a flow cytometry protocol as described [69]. Exponentially growing cells were pulsed with 1 μM BrdU (Sigma) for 36 hours and then fixed with methanol at −20°C. Cells were blocked in 3% BSA in PBS for 30 minutes and then stained with a FITC-labeled, anti-BrdU monoclonal antibody (Sigma, Anti-BrdU, B44) for 1 hour. Flow cytometry was performed on a Becton Dickinson LSR II instrument.

### Gemcitabine sensitivity

The sensitivity of Ewing sarcoma cell lines to gemcitabine, compared to other cancer cell lines, was assessed using data from the Genomics of Drug Sensitivity in Cancer resource (http://www.cancerrxgene.org/) [36].

### Apoptosis assays

Caspase-3/7 activation was measured using the Caspase-Glo 3/7 Luminescence assay (Promega), according to the manufacturer's instructions. Annexin V was measured using a FITC Annexin V/Dead Cell Apoptosis Kit (ThermoFisher). The flow cytometry data were analyzed using FlowJo.

### siRNA transfection

Cells (1.5-3 × 10) were plated one day prior to transfection in six-well plates. Cells were transfected with siRNA using Lipofectamine RNAiMax (ThermoFisher Scientific) according the manufacturer's instructions. siRRM1_pool, siRRM2_pool, and siSLFN11 were SMARTpool ON-TARGETplus reagents (GE Dharmacon). The sequences for si_RRM2_R2B and si_NT were 5′-GAUUUAGCCAAGAAGUUCAGA-3′ and 5′-UAGCGACUAAACACAUCAAUU-3′, respectively.

### Cloning of siRNA-resistant RRM2

The full length RRM2 cDNA, modified between base pairs 164-182 to 5′-CTACGGA ACCCAAAACGAA-3′, was obtained as a gene block (IDT) and inserted into the Tet-One vector (pTO; Clontech). After verification by sequencing, the plasmid (pTO-RRM2) was used to make lentivirus.

### Lentivirus production and infection

Lentivirus was produced by transfecting HEK-293T cells with the pTO-RRM2 plasmid and packaging plasmids (psPAX2 and pMD2.G) according to the FuGENE 6 (Roche) protocol. For the lentiviral transduction, Ewing sarcoma cells were incubated with 2 mL of virus and 6 μg/mL of polybrene (Sigma-Aldrich) for 12-16 hours. Cells were selected in 1 μg/mL puromycin 48 hours after transduction.

### Statistical analysis

Student's *t*-test was used to calculate p-values. Statistical analyses were conducted using GraphPad Prism 5.0.

## SUPPLEMENTARY INFORMATION FIGURES AND TABLES




