# Supplementary material for: Gene expression signature based screening identifies ribonucleotide reductase as a candidate therapeutic target in Ewing sarcoma
Source: Oncotarget. 2016 Aug 19;7(39):63003–19. doi: 10.18632/oncotarget.11416 (PMC5325343; doi:10.18632/oncotarget.11416)
Supplement: Supplementary file 1 [file oncotarget-07-63003-s001.pdf]

Gene expression signature based screening identifies ribonucleotide reductase as a candidate therapeutic target in Ewing sarcoma

Supplementary Information

MATERIALS AND METHODS

Protein isolation and immunoblotting: Whole-cell extracts for immunoblotting were prepared by incubating cells in RIPA buffer (Boston BioProducts) plus protease and phosphatase inhibitors (Halt Protease & Phosphatase Inhibitor Cocktail, EDTA-free. ThermoFisher Scientific) for 20 min. Supernatants were collected following a 15 min centrifugation at 17,000 r.c.f. at 4°C. Protein concentrations were determined using the BCA reagent (Pierce). SDS-PAGE was used to separate proteins, which were then transferred to polyvinylidene difluoride membranes (Millipore). Antibodies to the following proteins were used in the immunoblots: FLI1 (C-19, Santa Cruz, sc-356, 1:1000), SLFN11 (E-4, Santa Cruz, sc-374339, 1:500), cleaved caspase-8 (18C8, Cell Signaling, #9496, 1:1000), phospho-Histone H2A.X (Ser139, 20E3, Cell Signaling, #9718, 1:1000), phospho-Chk1 (Ser345, 133D3, Cell Signaling, #2348, 1:1000), phospho-Chk1 (Ser317, D12H3, Cell Signaling, #12302, 1:1000), Chk1 (2G1D5, Cell Signaling, #2360, 1:1000), PARP (46D11, Cell Signaling, #9532, 1:1000), RRM1 (Proteintech, 10526-1, 1:1000), RRM2 (Proteintech, 116611-1, 1:500), and tubulin (Proteintech, 66031-1, 1:2000).

Gene ontology analysis: Gene ontology analysis was performed using ToppGene (https://toppgene.cchmc.org/) [1].

Anchorage-independent growth assay: Cells (2×10^4) were plated in triplicate in 12-well plates in methylcellulose matrix (ClonaCell-TCS Medium, Stemcell Technologies) with ciclopirox (10 μM) or vehicle and incubated for ten days. Colonies were then counted using a Nikon inverted microscope.

dNTP quantification: dNTPs were quantified using a PCR-based protocol [2]. A673 and TC71 cells were cultured in the presence of ciclopirox or DMSO for 24 hours. The cells were then detached with trypsin, re-suspended in 10 ml of cold PBS, and counted using a Z2 Coulter Counter (Beckman Coulter). The cells were centrifuged for 5 min at 3000g at 4°C, the supernatant was discarded, and the cells were re-suspended in cold 60% methanol. The cells were then placed at 95°C for 3 min, followed by sonication for 30 s. The extracts were centrifuged to remove debris and the supernatants were passed through pre-equilibrated Amicon Ultra-0.5-ml centrifugal filters at 4°C to remove macromolecules >3 kDa. The filtrate was evaporated under centrifugal vacuum at 70°C and the pellet was then re-suspended in 25 μl of nuclease-free water. PCR was then performed using the protocol described by Wilson et al[2].

Cell cycle analysis: Cell cycle analysis was performed using the Click-iT EdU kit for flow cytometry (ThermoFisher Scientific). Cells were labeled with EdU for 2 hours and analysis was performed according to the manufacturer's instructions. Flow cytometry was performed on a Becton Dickinson LSR II instrument.

Transferrin and N-acetylcysteine rescue experiments: Holo-transferrin (Sigma) and N-acetylcysteine (Sigma) were added to the culture media at 10 μM and 2 mM concentrations, respectively. Cell viability was measured using CellTiter-Glo, as described.

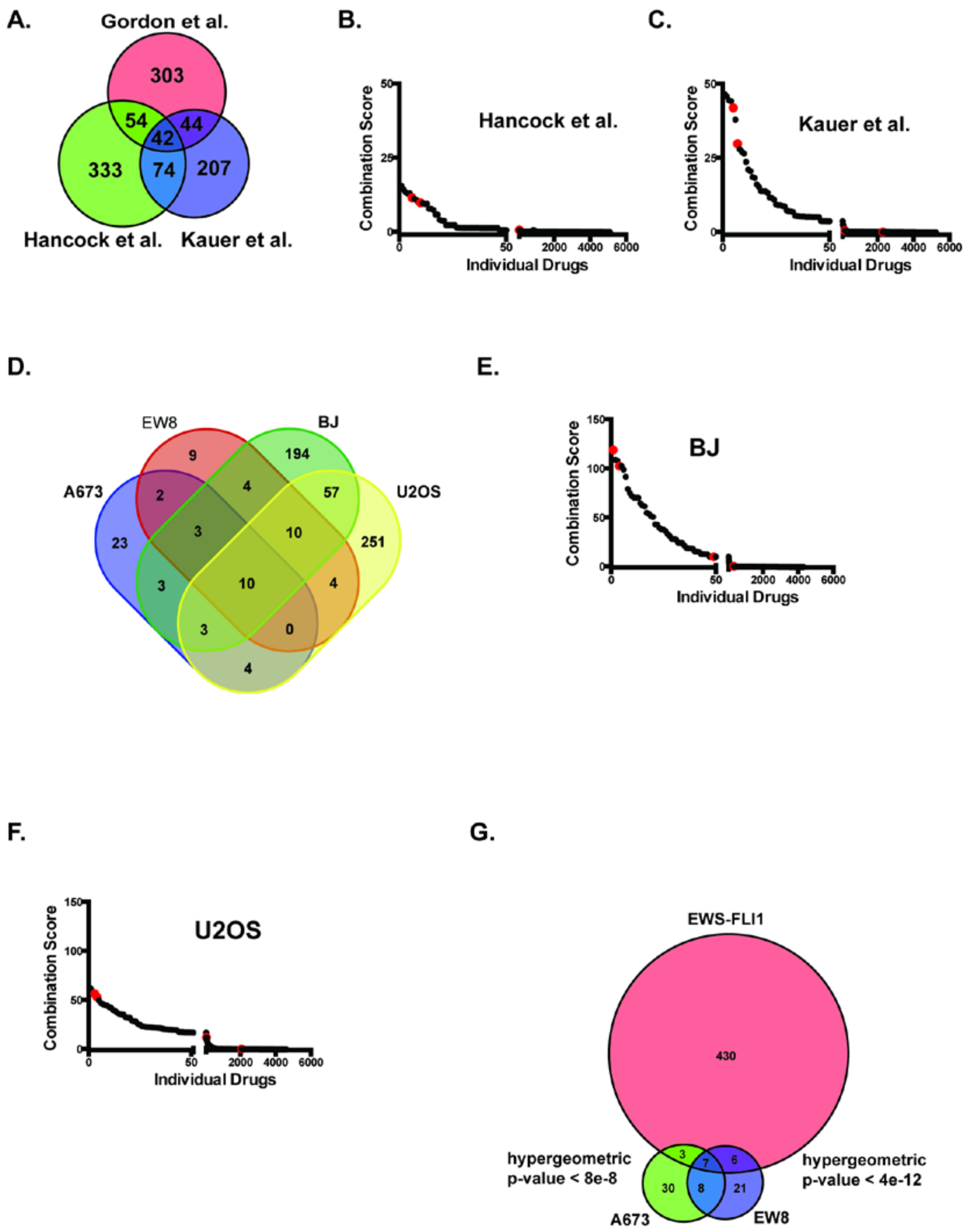

Supplementary Figure 1: Independent EWS-FLI1 data sets identify iron chelators as drugs that downregulate genes that are upregulated by EWS-FLI1. (A) Venn diagram showing the overlap between the Gordon et al., Hancock et al. and Kauer et al. EWS-FLI1 gene sets. (B, C) CMAP

was used to identify drugs that downregulate EWS-FLI1 target genes identified in the Hancock et al. (B) and Kauer et al. (C) analysis. The red dots represent ciclopirox. (D) Venn diagram showing the overlap between genes downregulated by ciclopirox in A673, EW8, BJ-tert and U2OS cells. (E) CMAP was used to identify drugs that downregulate genes downregulated by ciclopirox in BJ-tert cells. The red dots represent ciclopirox. (F) CMAP was used to identify drugs that downregulate genes downregulated by ciclopirox in U2OS cells. The red dots represent ciclopirox. (G) Venn diagram showing the overlap between EWS-FLI1 target genes identified by Gordon et al. and genes downregulated by ciclopirox in A673 and EW8 cells.

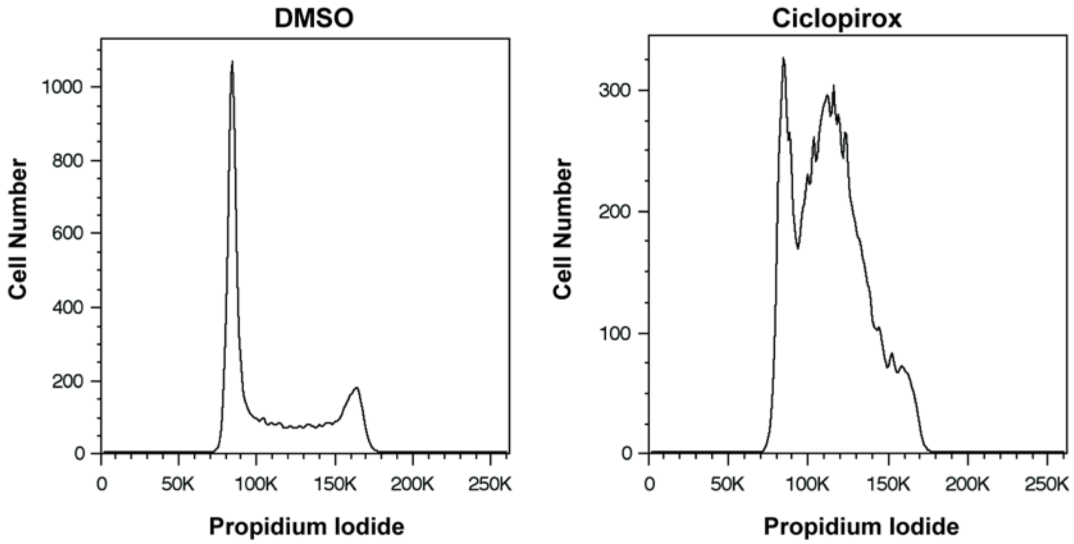

Supplementary Figure 2. Ciclopirox impairs S phase progression of Ewing sarcoma cells. A673 cells were treated with ciclopirox (10 μM) for 24 hours, fixed with methanol and then stained with propidium iodide.

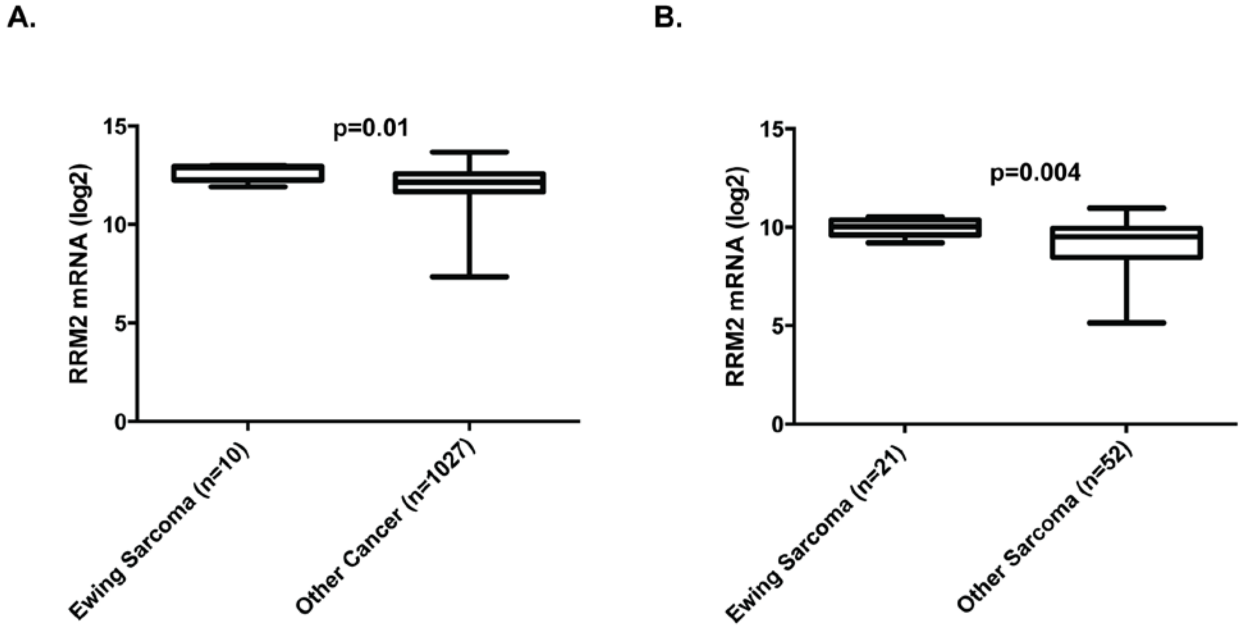

Supplementary Figure 3: RRM2 is highly expressed in Ewing sarcoma cell lines compared to non-Ewing sarcoma cancer cell lines. mRNA levels for RRM2 are shown for cancer cell lines in the (A) Cancer Cell Line Encyclopedia (http://www.broadinstitute.org/ccle/home) and the (B) National Cancer Institute Sarcoma Project (http://sarcoma.cancer.gov/sarcoma/).

A. B.

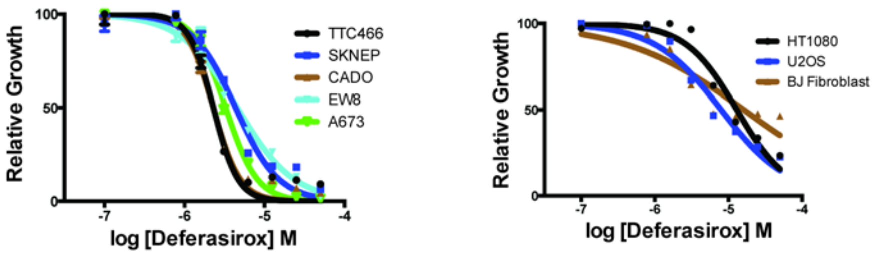

Supplementary Figure 4: The iron chelator deferasirox impairs the growth of Ewing sarcoma cells. (A) Dose-response curves for five Ewing sarcoma cell lines treated with different concentrations of deferasirox for three days. (B) Dose-response curves for non-Ewing sarcoma cancer cell lines treated with different concentrations of deferasirox for three days. Cell viability was assessed using the CellTiter-Glo Luminescent Assay. Results are representative of two independent experiments. Error bars represent mean ± SD of three technical replicates.

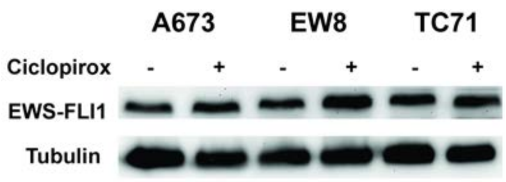

Supplementary Figure 5: Ciclopirox does not alter EWS-FLI1 levels. Western blot showing that ciclopirox treatment (10 μM) does not impact EWS-FLI1 levels in three Ewing sarcoma cell lines.

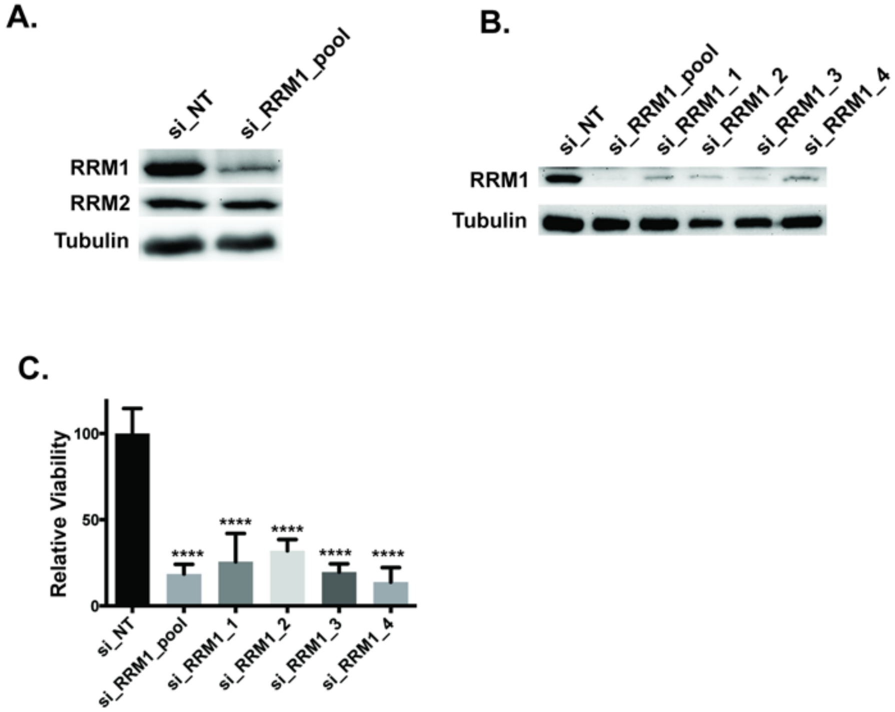

Supplementary Figure 6: Knockdown of RRM1 with siRNA impairs the viability of Ewing sarcoma cells. (A) Western blot showing that RRM2 levels are not affected by the si_RRM1_pool siRNA. (B) Western blot showing levels of the RRM1 protein after transfection with si_RRM1_pool or the individual siRNAs that compose the pool. (C) Relative viability of A673 cells transfected with si_NT, si_RRM1_pool and the individual siRNAs that compose the pool (1-way ANOVA, Dunnett's *post hoc* test). Cell viability was assessed using the CellTiter-Glo Luminescent Assay. Results are representative of three independent experiments. Error bars represent ± SD of three technical replicates. **** p-value < 0.0001.

Supplementary Table 1: Genes upregulated by EWS-FLI1 in the Gordon et al. data set.

For Table S1, please see the attached Excel file

Supplementary Table 2: Drugs that downregulate EWS-FLI1 target genes (adjusted p-value < 0.05).

For Table S2, please see the attached Excel file

Supplementary Table 3: Genes downregulated by ciclopirox in A673 and EW8 cells (Fold > 2 and FDR < 0.05).

For Table S3, please see the attached Excel file

Supplementary Table 4: Genes downregulated by ciclopirox in both A673 and EW8 cells (Fold > 2 and FDR < 0.05) and used for gene ontology analysis.

For Table S4, please see the attached Excel file

SUPPLEMENTARY REFERENCES

1. Chen J, Bardes EE, Aronow BJ, Jegga AG. ToppGene Suite for gene list enrichment analysis and candidate gene prioritization. Nucleic Acids Research. 2009; 37: 305–11.

2. Wilson PM, LaBonte MJ, Russell J, Louie S, Ghobrial AA, Ladner RD. A novel fluorescence-based assay for the rapid detection and quantification of cellular deoxyribonucleoside triphosphates. Nucleic Acids Research. 2011; 39(17): 112–2.
